# Non-sustained Ventricular Tachycardia as a Presentation of Arrhythmogenic Right Ventricular Cardiomyopathy

**DOI:** 10.7759/cureus.38620

**Published:** 2023-05-06

**Authors:** Adina Amin, Nadian Bailey, Amanda Warren, Bharath Reddy

**Affiliations:** 1 Internal Medicine, NewYork-Presbyterian Brooklyn Methodist Hospital, Brooklyn, USA; 2 Cardiac Electrophysiology, NewYork-Presbyterian Brooklyn Methodist Hospital, Brooklyn, USA

**Keywords:** arvd, nsvt, atrial tachyarrhythmia, non-sustained ventricular tachycardia, idiopathic dilated cardiomyopathy, arrhythmogenic right ventricular dysplasia

## Abstract

Arrhythmogenic right ventricular cardiomyopathy (ARVC) is a rare disorder with familial (autosomal dominant) predisposition and can be challenging to diagnose. Non-sustained ventricular tachycardia (NSVT) is a relatively uncommon and short-lived arrhythmia when seen in the general, healthy population. NSVT with a left bundle branch block morphology is usually idiopathic but may also be seen in ARVC. It can also be associated with poorer prognosis and increased mortality. Repetitive monomorphic ventricular ectopic beats may suggest ARVC, but could also be idiopathic. Timely diagnosis is vital due to the unpredictability and progressive nature of ARVC. We present a case of a 40-year-old Caucasian female with heart palpitations and NSVT found on an outpatient Holter monitor, and later found to have clinical and radiological features consistent with ARVC.

## Introduction

Arrhythmogenic right ventricular cardiomyopathy (ARVC), formerly known as arrhythmogenic right ventricular dysplasia, is a fairly new and intriguing disorder that was first described in 1982 by Marcus et al. [1]. Diagnosis of ARVC requires multiple tests including electrocardiogram (EKG), transthoracic echocardiogram (TTE), and cardiac computed tomography (CT). Diagnosis can also be made using additional workup, such as genetic testing, and cardiac magnetic resonance imaging (MRI). Patients with ARVC often have fibrofatty infiltration of their right ventricle and will have multiple EKG changes, such as a left bundle branch block or epsilon waves [2]. Presentation can range from asymptomatic ventricular ectopy to non-sustained ventricular tachycardia (NSVT), ventricular tachycardia (VT), or sudden cardiac death [2]. NSVT has been induced in up to 4% of asymptomatic adults, more commonly seen in men and with increasing age [3]. NSVT is more common in patients with a poor ejection fraction; up to 27.6% in patients with an ejection fraction of 35-50% [4]. According to a paper by Katritis et al., a commonly accepted definition of non-sustained ventricular tachycardia (NSVT) is “three (sometimes five) or more consecutive beats arising below the atrioventricular node with an RR interval of <600 ms (>100 beats/minutes) and lasting <30 seconds" [5].

## Case presentation

**Figure 1:**
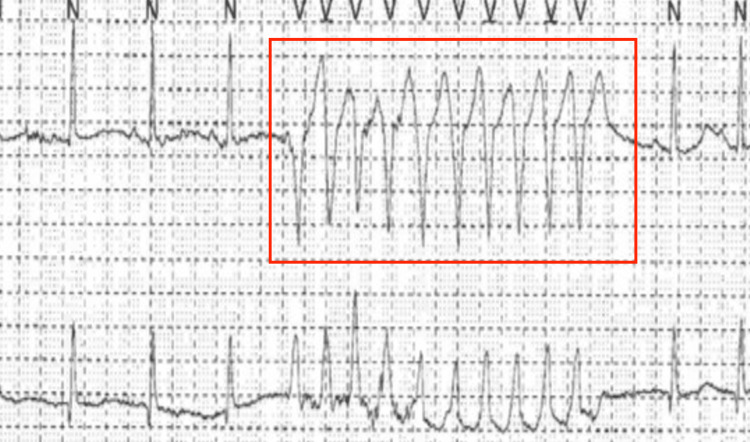
Holter monitor reading showing NSVT within the red box NSVT: non-sustained ventricular tachycardia

A 40-year-old Caucasian female with a past medical history of heart palpitations, anxiety, depression, and chronic pain due to a motor vehicle accident, presented to the emergency department after her primary cardiologist notified her that her Holter monitor showed an abnormality that necessitated admission. Her Holter monitor revealed two episodes of NSVT, each lasting 10 beats, shown in Figure 1.

**Figure 2:**
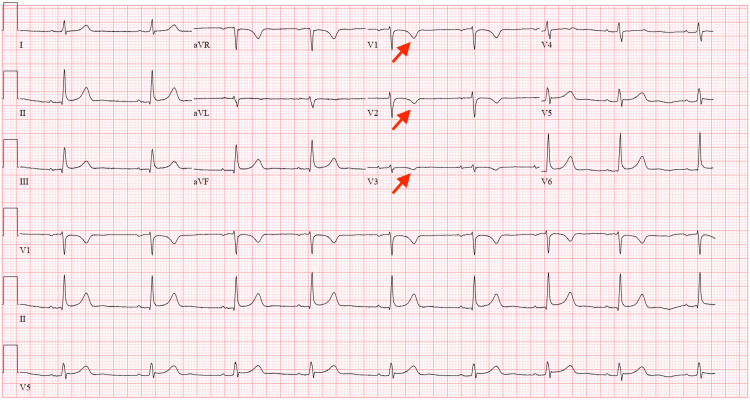
EKG revealed sinus bradycardia, with red arrows pointing to T wave inversions in V1, V2, and V3

She stated that her anxiety had been present for about a year after her motor vehicle accident, and has been associated with self-limited episodes of shortness of breath, heart palpitations, and dizziness. She kept a journal log of her palpitations. She denied a family history of sudden cardiac death and she denied any illicit drug use. On admission, she denied heart palpitations, chest pain, dizziness, recent illness, headache, or any other medical complaints. Her vital signs on admission were all within normal limits. Her heart rhythm was regular, normal rate, and there were no murmurs, rubs, or gallops auscultated. On physical exam, she was in no apparent distress, euvolemic, with clear lungs to auscultation. Her labs on admission were also unremarkable, including serum potassium 4.2 mmol/L, serum magnesium 2.2 ng/dL, troponin I <0.15 ng/mL, and thyroid stimulating hormone 1.23 micro IU/mL. EKG revealed T wave inversions in V1, V2, and V3, as seen in Figure 2. 

**Figure 3:**
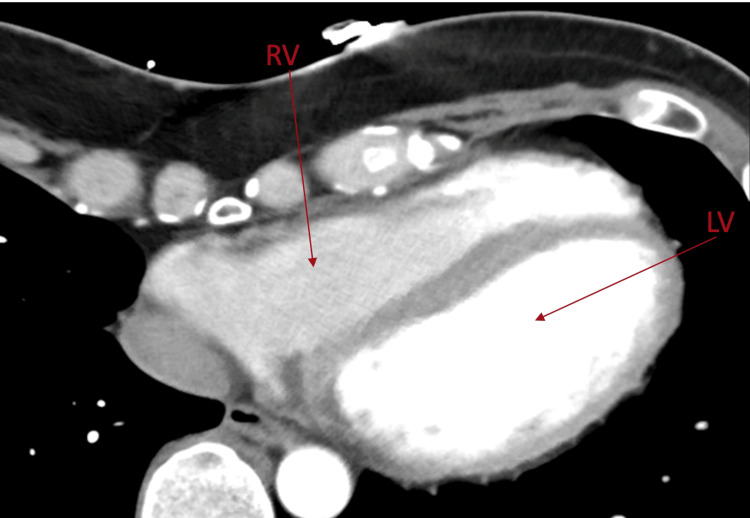
Cardiac CT showing the aneurysmal right ventricular apex, with arrows pointing to the right ventricle (RV) and left ventricle (LV)

She was admitted to the telemetry unit for close monitoring of her heart rhythm and electrolytes. A transthoracic echocardiogram showed an ejection fraction of 60%, with no regional wall motion abnormality, or valvular disease. A cardiac CT revealed a calcium score of zero, no significant coronary artery stenosis, a markedly elongated right ventricle, and an aneurysmal right ventricular apex. The dilated right ventricle and aneurysm are seen in Figure 3. 

The patient was discharged with a life vest, a referral for cardiac MRI for more accurate end-diastolic volume measurements, and genetic testing for prognostication. A cardiac MRI was obtained a few months later, which showed a mild outpouching of the right ventricular apex, which may indicate subtle remodelling sequela of arrhythmogenic right ventricular cardiomyopathy or other myopathic processes. There was a mildly dilated right ventricle with normal right ventricular systolic function, right ventricular ejection fraction of 56%, and no regional wall motion abnormalities. Additionally, there was no evidence of fibrofatty scar replacement or gadolinium enhancement.

The patient went through genetic testing due to findings suggesting Duchenne muscular dystrophy in both her son and her father. The patient tested positive for being a carrier of the Duchenne muscular dystrophy/Becker muscular dystrophy, *DMD* gene (DMD c1812+1G>A). 

Her cardiologist recommended an implantable cardioverter-defibrillator (ICD) or implantable loop recorder (ILR); however, she declined device implantation. Therefore, she was recommended to have a repeat cardiac MRI in one year and to seek medical attention for worsening symptoms. 

## Discussion

ARVC is an incredibly rare disorder (one in 5000 individuals) with an autosomal dominant inheritance pattern due to mutation of genes encoding the cardiac desmosome [2]. Presentation can be asymptomatic; however, some experience chest pain, palpitations, or shortness of breath. EKG can show T wave inversions in V1-V3 and epsilon waves. Other findings such as asymptomatic EKG changes, ventricular ectopy, non-sustained ventricular tachycardia, sustained ventricular tachycardia, or even sudden cardiac death can occur. Echocardiogram can show right ventricular wall motion abnormality or reduced ejection fraction, and a cardiac CT or MRI can show increased right ventricular end-diastolic volume.

According to the modified task force criteria for ARVC, there are three designations of ARVC: (i) definite (containing two major, or one major and two minor, or four minor criteria from different categories), (ii) borderline (one major and one minor or three minor criteria from different categories), and (iii) possible (one major or two minor criteria from different categories) [6]. The 2010 revised Task Force Criteria for ARVC include the following categories: global and/or regional dysfunction and structural alterations, tissue characterization of wall, repolarization abnormalities on the EKG, repolarization/conduction abnormalities on the EKG, arrhythmias, and significant family history [6]. The specifics for the major and minor criteria can be found in the 2010 revised Task Force Criteria for ARVC [6]. Our patient meets definite criteria given that she has two major criteria (right ventricular aneurysmal apex and T wave inversions) and one minor criteria (10 beats of NSVT on Holter monitor of unclear morphology).

Furthermore, there is a significant correlation between ARVC and Duchenne muscular dystrophy. They share histological, molecular and cellular pathogenic mechanisms, including muscle degeneration, inflammation and tissue replacement by fibrosis and fat [7]. Additionally, they are both genetically transmitted and involve common molecular pathways (Wnt and Hippo signalling) [7]. Thus, genetic testing becomes a crucial aspect for diagnoses of those with suspected ARVC. 

Patients with ARVC are generally advised to avoid high-intensity endurance exercise. Treatment is usually a beta blocker and possible implantable cardiac defibrillator placement (if the patient has a risk of cardiac arrest or sustained ventricular tachycardia). Poor prognostic factors include patients with multiple mutations, those with more severe left ventricular dysfunction, heart failure, cardiac transplant, and desmoplakin (DSP) mutation carriers [8].

## Conclusions

Although rare, ARVC is a potentially fatal genetic condition. High clinical suspicion of ARVC is essential for prompt diagnosis and improved prognosis. Physicians should obtain a meticulously detailed family and personal medical history for any patients with cardiac complaints such as heart palpitations, even in young patients. Catastrophic complications can potentially be avoided with close monitoring and preventive treatment (e.g. defibrillator placement, or even orthotopic heart transplantation in advanced cases of ARVC).
